# Loosening of Side-Chain Packing Associated with Perturbations in Peripheral Dynamics Induced by the D76N Mutation of β_2_-Microglobulin Revealed by Pressure-NMR and Molecular Dynamic Simulations

**DOI:** 10.3390/biom9090491

**Published:** 2019-09-16

**Authors:** Kazumasa Sakurai, Ryosuke Tomiyama, Takuma Shiraki, Yasushige Yonezawa

**Affiliations:** 1 High Pressure Protein Research Center, Institute of Advanced Technology, Kindai University, 930 Nishimitani, Kinokawa, Wakayama 649-6493, Japan; yonezawa-wk@waka.kindai.ac.jp; 2 Graduate School of Biology-Oriented Science and Technology, Kindai University, Wakayama 649-6493, Japan; 1933710005g@waka.kindai.ac.jp; 3 Department of Science and Technology on Food Safety, Faculty of Biology-Oriented Science and Technology, Kindai University, Wakayama 649-6493, Japan; shiraki@waka.kindai.ac.jp

**Keywords:** High pressure experiment, NMR, β_2_-microglobulin, MD simulation, amyloid fibril

## Abstract

β_2_-Microglobulin (β_2_m) is the causative protein of dialysis-related amyloidosis, and its D76N variant is less stable and more prone to aggregation. Since their crystal structures are indistinguishable from each other, enhanced amyloidogenicity induced by the mutation may be attributed to changes in the structural dynamics of the molecule. We examined pressure and mutation effects on the β_2_m molecule by NMR and MD simulations, and found that the mutation induced the loosening of the inter-sheet packing of β_2_m, which is relevant to destabilization and subsequent amyloidogenicity. On the other hand, this loosening was coupled with perturbed dynamics at some peripheral regions. The key result for this conclusion was that both the mutation and pressure induced similar reductions in the mobility of these residues, suggesting that there is a common mechanism underlying the suppression of inherent fluctuations in the β_2_m molecule. Analyses of data obtained under high pressure conditions suggested that the network of dynamically correlated residues included not only the mutation site, but also distal residues, such as those of the C- and D-strands. Reductions in these local dynamics correlated with the loosening of inter-sheet packing.

## 1. Introduction

β_2_-Microglobulin (β_2_m) is a light chain of the type I major histocompatibility antigen (MHC-1) with 99 amino acid residues. β_2_m is the causative protein of dialysis-related amyloidosis, a serious complication for patients receiving hemodialysis for more than ten years [1,2]. In the last few decades, the structural properties of β_2_m amyloid fibrils and the physicochemical mechanisms underlying fibril formation have been extensively examined [1,3,4,5,6,7].

**Figure 1:**
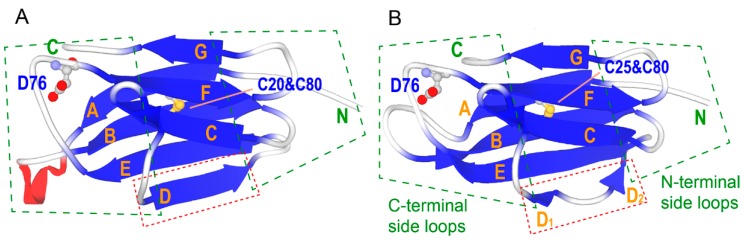
Crystal structures of β_2_m in an isolated monomer (**A**, Protein Data Bank (PDB) ID: 2yxf [8]) and in the major histocompatibility antigen (MHC) class I complex (**B**, PDB ID: 1hsb [9]). The broken green boxes indicate the N- and C-terminal side loop regions. The broken red boxes indicate the D-strand, which shows a prominent polymorphism. The side chains of D76, C25, and C80 are depicted as balls and sticks.

β_2_m consists of seven β-strands (A-G). Strands A, B, E, and D form one β-sheet, while strands C, F, and G form another sheet (Figure 1). These two sheets are linked by the disulfide bond between Cys25 and Cys80. Since all strands are antiparallel, inter-strand loops appear alternatively on either side of the sheet. Here, loops on the same side of the N terminus of the β_2_m molecule are called “N-terminal side loops”, whereas those on the opposite side are called “C-terminal side loops” (Figure 1, broken green boxes).

Although the overall structures of β_2_m elucidated under various conditions by X-ray crystallography or NMR are similar, some local polymorphisms have been reported. The D-strand mainly assumes two alternative conformations. In the structures of an isolated β_2_m molecule, the D-strand forms a regular antiparallel hydrogen bond registration with the adjacent E-strand (Figure 1A) [8,10,11], whereas the β_2_m portion in major histocompatibility complex I has a bulged structure on the middle region of the D-strand (Figure 1B) [9]. The NMR structure solved at pH 6.6 also shows a bulged structure [12]. Comparisons of these structures indicated that the β_2_m molecule contains inherent polymorphisms at some regions, e.g., the AB loop, CD loop, D-strand, and DE loop (Supplementary Materials, Figure S1) [8]. 

An aggregation-prone variant of β_2_m, D76N β_2_m, has also been reported. This is a naturally occurring variant that was discovered in members of a French family who had progressive bowel dysfunction with extensive visceral amyloid deposits composed of β_2_m [13]. The D76N mutant was found to be less stable than the wild type (WT) and fibril formation by D76N β_2_m was primed by exposure to a hydrophobic-hydrophilic interface under physiological intensity shear flow [14]. Since the crystal structures of WT and D76N appear to be similar to each other (Supplementary Materials, Figure S1), enhanced amyloidogenicity may be attributed to structural changes in hidden states behind the native state or modified structural dynamics [15,16]. A number of NMR studies and/or molecular dynamic (MD) simulations have been conducted in order to elucidate the mechanisms underlying the enhanced amyloidogenicity of the D76N mutant. The majority of findings obtained showed the destabilization of edge strands (i.e., A- and D-strands) and subsequent exposure of internal hydrophobic regions [15,16]. Some studies suggested that the D76N mutation enhances dimerization, which appears to be necessary for fibril formation [16,17,18,19]. However, other studies indicated that the mutation induces a consolidation of the D-strand, giving a rigid edge to the β-sheet and elongation end to the fibril [20].

We focused on the relationship between the effects of the D76N mutation and changes in the stability and dynamics of the β_2_m molecule. In order to obtain information on the hidden dynamics behind the native structure, we employed pressure because it enables access to these hidden states by shifting the conformational equilibrium [21]. Furthermore, among various denaturation experiments, analyses of pressure-induced conformational transitions provided distinct information, such as changes in molar volumes and conformational fluctuations upon transition at the amino acid residue level [22,23]. We applied pressure to induce structural transitions in wild-type and D76N β_2_ms and analyzed the changes induced using NMR and MD simulations to clarify the effects of the mutation at the atomic level. 

NMR measurements showed changes in residue-specific conformational changes upon the mutation or application of pressure. The MD simulation reproduced molecular motion consistent with NMR measurements and provided more detailed information on conformational changes. These results indicated that the mutation induced the loosening of the core packing of the β_2_m molecule, which is relevant to the destabilization of the whole molecule and subsequent enhancements in amyloidogenicity. Furthermore, the mutation induced decreases in specific fluctuations at some peripheral residues, including the D-strand and mutation site, which are inherently harbored in the native β_2_m molecule. The application of pressure was also found to suppress local dynamics. Reductions in local dynamics appeared to correlate with the loosening of inter-sheet packing.

## 2. Results

### 2.1. Selection of Experimental pH Conditions

The native state of β_2_m under physiological conditions is too stable to show pressure-induced conformational transitions within the accessible pressure range. Thus, prior to the main experiments, we identified pH conditions under which the native conformation is slightly destabilized. We measured the pH-dependent tryptophan fluorescence of β_2_m molecules (Supplementary Materials, Figure S2), giving clear unfolding transitions. Data were fit with sigmoidal curves and the midpoint pH values of unfolding were 4.11 ± 0.04 for the wild type and 4.54 ± 0.03 for D76N β_2_m. These results showed that the D76N is less stable than WT, being consistent with previous reports [13]. Based on these results, the following pressure experiments were conducted at pH 5.0 for the wild type and pH 5.5 for D76N, namely, pH values one unit higher than the midpoints.

We examined protein dynamics for WT and D76N under ambient and high-pressure conditions. Hereafter, the combinations of the measurement conditions are denoted as WT(AP), WT(HP), D76N(AP), and D76N(HP). Standard conditions are WT(AP). The difference between the results obtained at WT(AP) and D76N(AP) was attributed to the effect of the mutation on the structure or dynamics of the β_2_m molecule, whereas that between the results at WT(AP) and WT(HP) was attributed to the pressure effect.

### 2.2. NMR Measurements for the Effect of the Mutation

**Figure 2:**
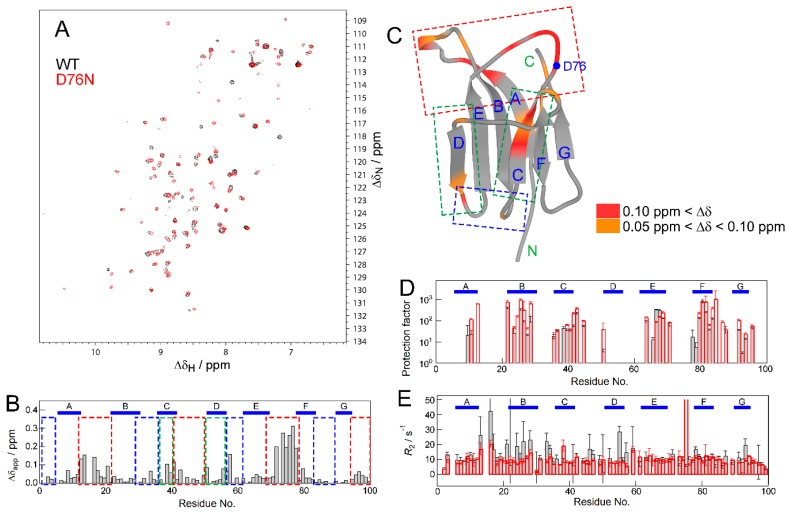
Comparison of NMR data of the wild type and D76N mutant. (**A**) An overlay of the ^1^H–^15^N HSQC spectra of the wild type (WT) (black) and D76N (red) under ambient pressure conditions. (**B**) A plot of apparent chemical shift differences (∆δ_app_) against residue numbers. (**C**) The mapping of residues with significant ∆δ values on the crystal structure. Colors indicate ∆δ values as indicated by the color gauge below. In panels B and C, the red and blue boxes indicate N- and C-terminal loop regions, respectively. The green box indicates the position of the C- and D-strands. (**D**) Protection factors for each residue obtained from H/D exchange experiments. The position without a bar indicates the residue that was exchanged too fast to be measured even in the first measurement of the HSQC spectrum. (**E**) The plot of *R*_2_ rates. Data indicated by gray and red bars in panels D and E are of WT and D76N, respectively.

We measured ^1^H–^15^N HSQC spectra for WT at ambient pressure (WT(AP)) and D76N at ambient pressure (D76N(AP)) (Figure 2A). Apparent chemical shift differences (∆δ_app_) for individual residues were then calculated using Equation (2) (see Methods) and residues with high ∆δ values were mapped on the crystal structure (Figure 2B,C). Significant ∆δ values were observed on most of the C-terminal side loop residues (red boxes), some N-terminal side loop residues (blue boxes), and some C- and D-strand residues (green boxes). Although the experimental pH condition for WT was slightly different from that of the mutant, we confirmed that the obtained ∆δ_app_ values certainly came from the mutation effect, by measuring a HSQC spectrum for WT at pH 5.5 and comparing it with the two spectra presented above (see Supplementary Materials, Figure S3). Since the mutation point (D76) is located on the C-terminal side of the molecule, the effect of the mutation was assumed to be transmitted to the opposite, N-terminal side of the molecule through the C- and D-strands.

To investigate the difference in local structural dynamics between WT and D76N, we conducted the hydrogen/deuterium (H/D) exchange experiment and *R*_2_ measurements (Figure 2D,E). Since the H/D exchanges of many residues were too fast to be observed under experimental pH, measurable protection factors were only obtained for the core residues. The distributions of the core residues were indistinguishable from each other (Figure 2D). On the other hand, the *R*_2_ profile of WT (Figure 2E) showed higher *R*_2_ values at some regions, such as the AB loop, B-strand, CD loop, and D-strand, than D76N, indicating that WT has more fluctuations at these residues. As described above, the wild-type β_2_m molecule has polymorphisms, particularly on the D-strand region. Therefore, the observed fluctuation in WT is caused by structural heterogeneity related to the polymorphism, and this fluctuation is reduced by the mutation. However, since limited information is currently available on the effects of the D76N mutation, we performed pressure experiments.

### 2.3. NMR Measurements for the Effect of Pressure

**Figure 3:**
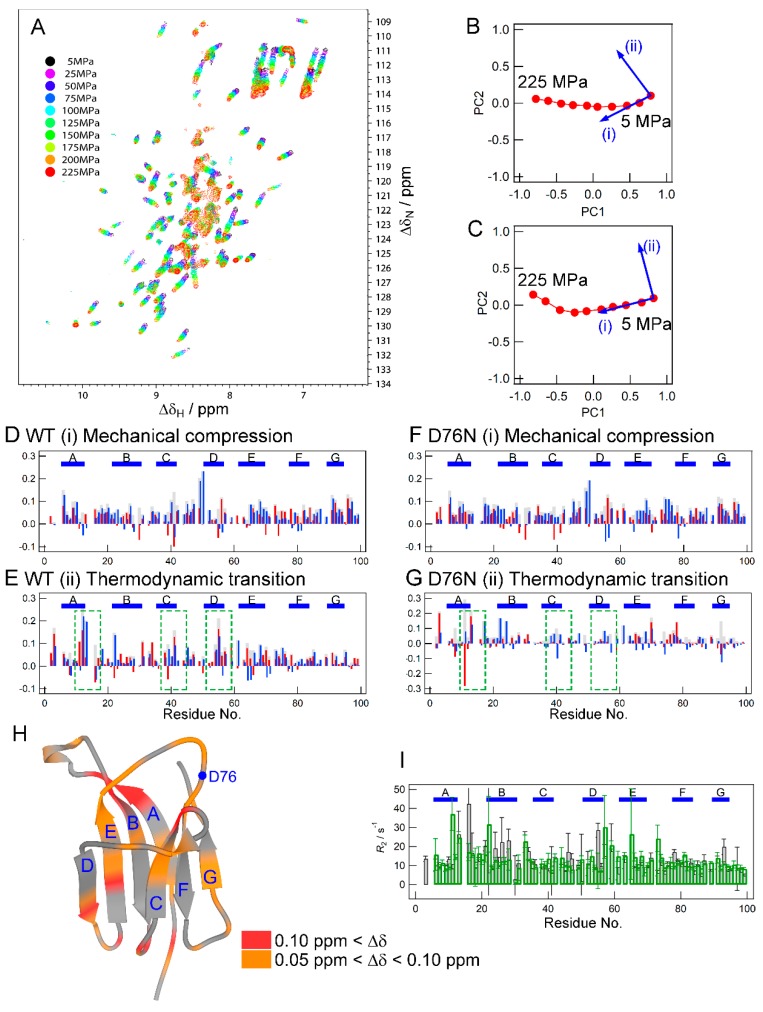
Data analyses of high-pressure NMR measurements. (**A**) Overlay of the ^1^H–^15^N HSQC spectra of wild-type β_2_m obtained at pressures ranging between 5 and 225 MPa. Spectral colors indicate the hydrostatic pressures at which measurements were performed. (**B**,**C**) The principal component (PC) planes of the pressure-dependent spectral changes for WT (**B**) and D76N (**C**) obtained from the singular value decomposition (SVD) procedure. A point on the plane corresponds to one spectrum. The rightmost point corresponds to the spectrum obtained in the initial conditions (5 MPa). The blue arrows indicate the supposed directions of the conformational changes corresponding to (i) mechanical compression and (ii) thermodynamic transition. (**D**–**G**) Calculated ∆δ patterns for (i) mechanical compression and (ii) thermodynamic transition for WT (**D**,**E**) and D76N (**F**,**G**), respectively, from the PCA-based analysis. Blue and red lines show chemical shift changes (in ppm) in the ^15^N and ^1^H directions, respectively. The ∆δ values for ^15^N were divided by 5. The gray bars are the ∆δ_app_ values calculated by Equation (2). (**H**) The mapping of residues with significant ∆δ values in the (ii) thermodynamic transition for WT (panel E) on the crystal structure. Colors indicate the ∆δ values as shown by the color gauge below. (I) Comparison of the *R*_2_ profile of wild-type β_2_m under ambient pressure conditions (condition WT(AP), gray bars) with those at 100 MPa (condition WT(HP), green bars).

It is reported that the application of pressure alters the conformational equilibrium, by which an undetectable minor state at ambient pressure becomes spectroscopically observable [21]. Thus, we applied high pressure to reveal the hidden property relevant to the mutation-induced structural change. The application of pressure to wild-type β_2_m caused continuous changes in HSQC signals (Figure 3A and Supplementary Materials, Figure S4A). ∆δ data contained information of the structural difference between WT(AP) and WT(HP). It is supposed that there are two contributions of high pressure to proteins: (i) mechanical compression and (ii) thermodynamic transition. The former corresponds to the general compression within a subensemble of the conformer (e.g., the basic native state) and the latter corresponds to the shift of conformational equilibrium between other subensembles (e.g., the native and intermediate states) characterized with different free energy levels [21,22]. We separated the ∆δ data into these two contributions by using the principal component analysis (PCA)-based procedure previously suggested [24,25]. Details of the data process are described in Supplementary Materials (Figure S4A,C,E,G, and its figure caption). As a result of a singular value decomposition (SVD) process, nine PCs were obtained and the first two PCs were judged to be relevant to the conformational changes. Figure 3B shows a plot of PC1 and PC2. Although the major spectral change is in the direction of PC1, a significant contribution of PC2 was also observed, indicating that the spectral change includes contributions from the two types of conformational changes: (i) mechanical compression and (ii) thermodynamic transition [21,25]. The spectral change at lower pressure regions is often attributed to mechanical compression in the case of a structured protein [21]. Thus, we set the direction along which the initial spectral change occurred as (i) mechanical compression (a blue arrow denoted as (i) in Figure 3B). On the other hand, (ii) thermodynamic transition generally occurs in a certain pressure range. Thus, the direction of the spectral change observed in the middle pressure region is considered to represent thermodynamic transition (ii) and we assumed that its contribution is orthogonal to the direction corresponding to (i) mechanical compression. Based on the directions identified, we calculated the ∆δ patterns corresponding to the directions of vectors (i) (Figure 3D) and (ii) (Figure 3E) for WT. The same measurements and analyses were performed on D76N (i.e., structural change from D76N(AP) to D76N(HP)) (Supplementary Materials, Figure S4B,D,F,H). The scores for PC1 and PC2 were shown in Figure 3C. The ∆δ patterns obtained for (i) and (ii) are shown in Figure 3F,G, respectively.

The ∆δ patterns of D76N for (i) mechanical compression were similar to those of WT (Figure 3D,F). On the other hand, the ∆δ patterns for (ii) thermodynamic transition were distinct from each other (Figure 3E,G). In the case of WT, pressure-dependent conformational changes were observed, particularly on the A-, C-, and D-strands (Figure 3E, green boxes and Figure 3H). These residues also showed significant ∆δ values upon the mutation (Figure 2B,C), indicating that pressure exerted similar effects on the β_2_m structure and dynamics as the D76N mutation. In combination with the MD simulation, we will discuss what kind of conformational changes are included on these regions (see Section 2.5). On the other hand, pressure induced less significant ∆δ values at the same regions in D76N (Figure 3G). These results further support the hypothesis that WT inherently has structural fluctuations at these residues. These fluctuations may also be suppressed by pressure. In contrast, D76N had less fluctuations in the corresponding regions, even under ambient pressure. Thus, there were few pressure-induced chemical shift changes (Figure 3G, green boxes). The pressure-induced suppression of the fluctuation observed in WT was also supported by the results of *R*_2_ measurements (Figure 3I), in which some residues showed decreases in their *R*_2_ values upon the application of pressure.

### 2.4. Results of MD Simulations

**Figure 4:**
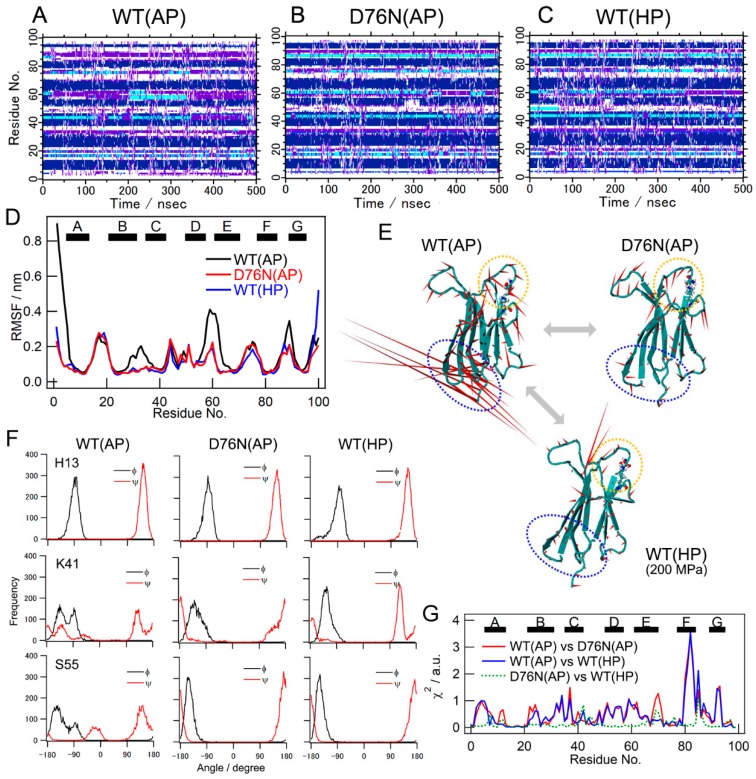
Results of molecular dynamic (MD) simulations. (**A**–**C**) Time evolution of the secondary structure profile observed under condition WT(AP): WT at ambient pressure (**A**), condition D76N(AP): D76N at ambient pressure (**B**), and condition WT(HP): WT at 200 MPa (**C**). Individual colors indicate the secondary structures according to the DSSP definition; blue: isolated β-bridge, deep blue: extended strand, cyan: hydrogen-bonded turn, purple: bend. (**D**) Root mean square fluctuations (RMSFs) calculated for individual simulation conditions. The black, red, and blue lines show RMSF for conditions WT(AP), D76N(AP), and WT(HP), respectively. (**E**) Visualizations of the dominant motions under individual simulation conditions. The first eigenvector values calculated by PCA of MD data are represented as dominant motions. The directions of the residues observed in the first PC were indicated by the red spikes. The blue and orange broken circles indicate the positions of the DE loop and mutation site, respectively. (**F**) The distribution of the dihedral angles of the representative residues, His13, Lys41, and Ser55 under conditions WT(AP), D76N(AP), and WT(HP). (**G**) χ^2^ values as an indicator of the degree of the difference between dihedral angle distributions under individual simulation conditions.

In order to clarify the structural and dynamical events occurring at the atomic level upon the mutation or application of pressure, we conducted MD simulations. The simulation period for individual conditions was 500 nsec. The time-dependent root mean square deviations have reached a plateau in the early stage of the simulation period, indicating that the simulated conformations were quickly equilibrated and converged around the stable, native structure (Supplementary Materials, Figure S5). Panels A, B, and C in Figure 4 showed the time evolution of the secondary structure under conditions WT(AP), D76N(AP), and WT(HP), respectively. Although the secondary structures of some loop regions, for example, around 44, 60, and 77, fluctuated between several types of secondary structures, structured regions consisting of β-sheets were basically maintained throughout the simulation periods.

We initially examined changes in overall motion. Figure 4D shows the root mean square fluctuations (RMSFs) of individual residues under respective conditions. As expected, all loop regions showed significant fluctuations under standard conditions (condition WT(AP): WT at ambient pressure). Among these loop regions, the N-terminal side loops, i.e., the N terminus and BC, DE, and FG loops, showed significant decreases in their fluctuations upon introducing the D76N mutation (Figure 4D, red trace) or applying pressure (blue trace), and the resultant profiles of the fluctuations were similar to each other. To visualize molecular motion under the respective conditions, we performed PCA on the MD structures, and compared the first PC motions as dominant motions (Figure 4E). Under condition WT(AP) (WT, ambient pressure), the N terminus, the loop region around D76 (the EF loop, indicated by an orange circle), and residues around the D-strand and the DE loop (indicated by the blue circle) showed large swaying motions, whereas residues consisting of β-sheets showed only slight motion. These motions consisted of a bending motion of the overall molecule. On the other hand, in the motions extracted under conditions D76N(AP) and WT(HP), these strong correlations disappeared. Reductions in fluctuations were qualitatively consistent with NMR results (see Figure 2E and Figure 3I).

We then inspected the distributions of the dihedral angles of all residues for residue-specific conformational changes (Figure 4F). For example, the φ and ψ angle distributions of K41 and S55 under condition WT(AP) were distinct from those obtained under condition D76N(AP) or WT(HP) (Figure 4F, middle and lower rows). On the other hand, the φ and ψ distributions of H13 were not dependent on the conditions (upper row). We herein calculated the χ^2^ values of individual residues using Equation (1) for the degrees of difference in dihedral-angle distributions.

(1) $$\chi^{2}=\frac{\sum_{j=1}^{n}\sum_{i=1}^{n}{\left(f_{1}\left(\varphi_{i},\psi_{j}\right)-f_{2}\left(\varphi_{i},\psi_{j}\right)\right)}^{2}}{n^{2}}$$

where *f*_1_ and *f*_2_ are the frequencies of a specified dihedral angle under the respective conditions, *n* is the number of bins, and the bin size was set to 36°; therefore, *n* = 10. Figure 4G shows the calculated χ^2^. The mutation (condition WT(AP) vs. D76N(AP), red trace) and application of pressure (WT(AP) vs. WT(HP), blue trace) induced significant changes in the dihedral angle distribution of many residues. These changes were similar to each other (Figure 4G, green dotted trace), indicating that the main chain conformation of WT under high pressure (WT(HP)) was similar to that of D76N at ambient pressure (D76N(AP)). However, the profiles obtained (Figure 4D,G) were not directly related to those obtained from NMR measurements (Figure 2 and Figure 3). Differences in the φ and ψ angles may not reflect ∆δ or relaxation profiles in NMR measurements. Thus, further analyses were performed.

### 2.5. Pressure- and Mutation-Induced Changes in Intramolecular Correlated Motions

**Figure 5:**
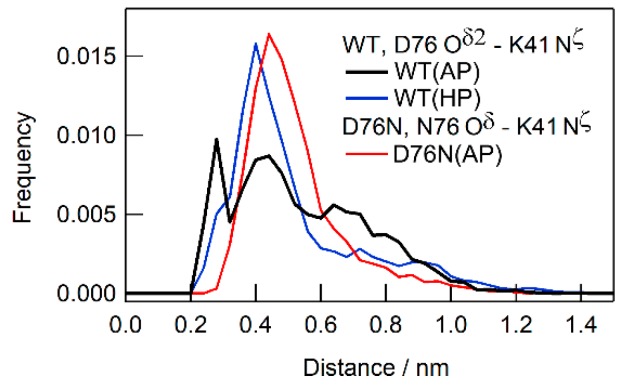
Distribution of distance between D76 and K41 side chains for WT at ambient pressure (WT(AP), black) and at 200 MPa (WT(HP), blue), respectively, while that between N76 and K41 side chains for D76N at ambient pressure (D76N(AP)) is indicated by a red line.

We examined local dynamics around the mutation site. According to the crystal structure, an electrostatic interaction occurred between the side chains of D76 and K41 [8]. The D76N mutant has a similar arrangement of the side chains of N76 and K41 [13]. We investigated the distributions of distance between the side chains, specifically between the O^δ2^ atom of D76 and N^ζ^ atom of K41 (Figure 5). At ambient pressure, WT was found to have three subensembles in the distance distribution, indicating that there are three distinct local interaction modes (condition WT(AP): Figure 5, black line). On the other hand, the distance distribution of the corresponding pair (O^δ^ atom of N76 and N^ζ^ atom of K41) of D76N under ambient pressure converged to a single peak (D76N(AP): Figure 5, red line), indicating that only one conformational ensemble was chosen. The application of pressure to WT also induced a similar convergence in distribution (WT(HP): Figure 5, blue line), also indicating that the application of pressure and the mutation induced similar conformational changes.

**Figure 6:**
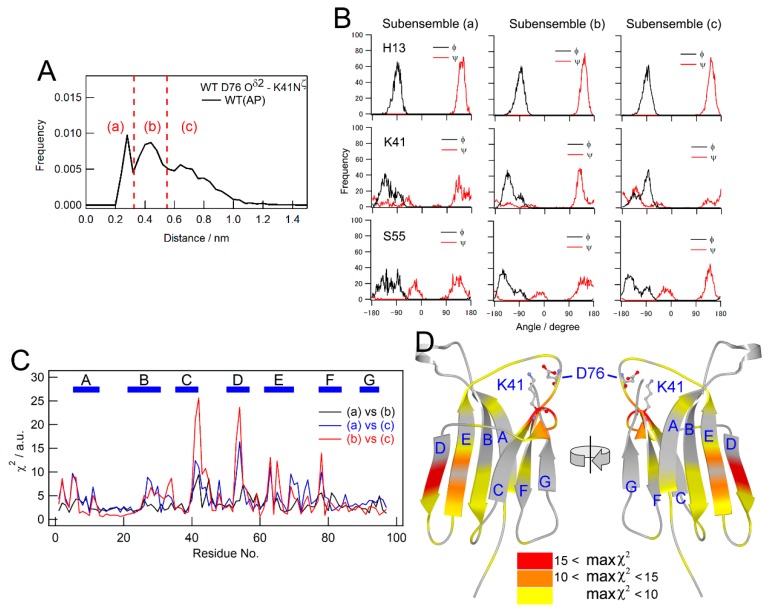
Correlation of dynamics between main chain conformations for all residues and the mutation site. (**A**) The distribution of distance between D76 and K41 side chains for WT at ambient pressure. The dotted lines are the borders separating the three subensembles, named (a), (b), and (c), of the conformation with different side chain distances. (**B**) The dependence of the dihedral angles of the representative residues (H13, K41, and S55) on the distance between the D76 and K41 side chains. (**C**) χ^2^ values as an indicator of the degree of the dependence of the dihedral angle distribution on the D76-K41 side chain distance. (**D**) The mapping of max χ^2^ values among those obtained for the three combinations ((a) vs. (b), (a) vs. (c), and (b) vs. (c)) on the crystal structure. Colors indicate χ^2^ values, as shown by the color gauge below.

We then speculated that correlations exist between the main chain conformations of individual residues and the local conformation around D76. To assess this hypothesis, we labeled each subensemble of the D76-K41 side-chain distance as (a), (b), and (c) (Figure 6A), and classified MD structures according to which subensemble the structure belongs to. The dihedral angle distribution of each residue was subsequently recalculated for individual subensembles (Figure 6B). For example, H13 showed a similar dihedral angle distribution irrespective of the D76-K41 side-chain distance, indicating no correlation with the D76-K41 distance (Figure 6B, upper row). On the other hand, the dihedral angle distribution of K41 strongly depended on the D76-K41 side-chain distance (middle row). This was expected because K41 is involved in the electrostatic interaction. However, other remote residues, e.g., S55, also showed the dependence of its dihedral angle distribution on the D76-K41 side-chain distance (lower row). Differences in dihedral angle distributions depending on which subensemble the sample belongs to were also assessed by calculating χ^2^ (Equation (1)). The results obtained are shown in Figure 6C. Some residues showed significant χ^2^ values, indicating that the conformation of these residues correlated with the local conformation around the mutation site. These residues were distributed on some remote residues from the mutation site, particularly the residues on the C-, D-, and E-strands (Figure 6D). An important result is that these residues were consistent with those found to be susceptible to the mutation (Figure 2B) or application of pressure (Figure 3E) through NMR measurements. These results also indicate that these residues are involved in the conformational heterogeneity of the native structure suggested above, and comprise the local dynamic network.

### 2.6. Pressure- and Mutation-Induced Loosening of the Inter-β Sheet Packing

**Figure 7:**
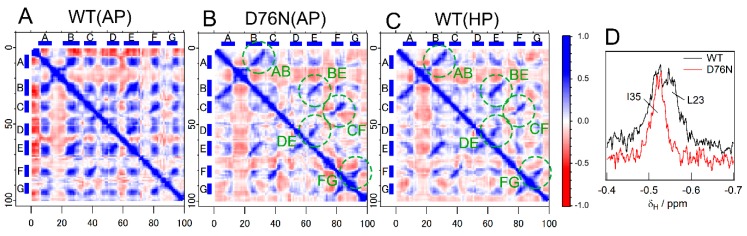
Assessment of inter-sheet packing. (**A**–**C**) Dynamic cross correlation matrices (DCCMs) calculated from MD results executed under conditions WT(AP) (**A**), D76N(AP) (**B**), and WT(HP) (**C**). The blue and red dots mean positively and negatively correlated motions, respectively, between the corresponding residues. (**D**) 1D ^1^H NMR data as a probe for inter-sheet packing. The black and red lines are the 1D ^1^H NMR spectra of WT and D76N at a high-field region, respectively.

The mechanisms underlying destabilization by the D76N mutation remain unclear. To obtain insights into the destabilization mechanism, we calculated the dynamic cross correlation matrix (DCCM) (Figure 7A–C). The DCCM presentation shows the degree of motional correlation between two residues. On the figure, the residue pairs whose motions were more likely to be parallel, anti-parallel, and with no correlated motion were colored blue, red, and white, respectively [26]. Regarding WT at ambient pressure (condition WT(AP)), the majority of the residues in β-strands showed a strong positive correlation (Figure 7A), indicating tight packing and subsequent collective motions of the two β-sheets. On the other hand, correlations in motion under conditions D76N(AP) and WT(HP) were less prominent (Figure 7B,C). Only residue pairs responsible for inter-strand hydrogen bonds in individual β-sheets (indicated by broken circles in B and C) maintained a strong correlation, indicating that each β-sheet was still stable, whereas inter-sheet packing loosened in the mutant (condition D76N(AP)) or WT under high pressure (condition WT(HP)). The loosening of side chain packing by the D76N mutation was confirmed based on 1D ^1^H NMR measurements of the high-field region (Figure 7D): at this region, signals from the methyl protons of L23 were observed for the wild type because of the ring current effect of the π electron of the Phe70 side chain under tightly packed conditions (Supplementary Materials, Figure S6). However, the corresponding signal of the D76N mutant was not observed, indicating the loosening of side chain packing of the D76N molecular structure.

## 3. Discussion

### 3.1. Similar Effects on Protein Conformation and Dynamics by the Mutation and Pressure

We investigated the destabilization mechanism of the D76N mutation on β_2_m using high-pressure measurements. The results obtained showed that the mutation induced reductions in the local dynamics of some peripheral residues, indicating that these reductions in dynamics are relevant to destabilization. Another result was that the effects of pressure on protein dynamics were similar to those of the mutation, indicating that the application of pressure and the mutation exerted common effects on the β_2_m structure. This situation enabled us to precisely investigate the mutation effect on protein dynamics by analyzing pressure-induced conformational changes.

An example of the similarity between pressure- and mutation-induced conformations was the ubiquitin Q41N mutant [27,28]: Kitazawa and co-workers designed this mutation to stabilize the pressure-induced conformation, named N2, based on the solved N2 structure. They concluded that the breakage of the hydrogen bond between Q41 and I36 is a key event for inducing a transition from the ground state to the N2 state. However, few studies have demonstrated that reductions in mobility and destabilization are coupled. The significance of the present results is that this coupling was detected via a pressure-induced population shift that mimicked the mutational shift in the population.

### 3.2. Mechanism of Destabilization by the Mutation

A similar consolidation of the D-strand induced by the D76N mutation was also reported by Chandrasekaran and Rajasekaran [20] in their MD simulations, confirming that the mutation decreased fluctuations around D76 as a result of reduction in frustration of the D-strand. However, reductions in local fluctuations were coupled with destabilization of the whole molecule. There are two possible explanations for destabilization. One is that local fluctuations confer the tight registrations of interactions to other regions. The suppression of inherent mobility in the wild-type structure may lead to the loss of the integrity of the interaction registration at different positions, particularly at the core region in this case. Another possibility is entropic compensation [29,30]: it is assumed that these correlated fluctuations are entropically contributing to the free energy of the immunoglobulin structure. However, the D76N mutation alleviated local fluctuations and incidentally reduced their mobility, generating internal fluctuations for entropic compensation.

### 3.3. Network of Local Dynamics

The present MD simulations showed that the region around D76 assumes multiple conformations (Figure 5), again indicating some structural frustration at this region. There may be a number of competitive conformation-determining interactions, such as the salt bridge between K41 and D76 side chains and hydrogen bonds between T73 and D76 and between Y78 and T73, as observed in the X-ray structure of D76N [13]. In the conformation of WT, several conformations are energetically comparable to each other and co-exist, leading to a specific fluctuation around the D76 region (Figure 5, black line). Crystal packing may bias one of the possible conformations in the X-ray structure. The D76N mutation abolishes the salt bridge, causing the disappearance of some possible conformers in the wild type and one conformation may be settled by specific interactions (Figure 5, red line). The application of pressure (Figure 5, blue line) induced a similar suppression of fluctuations possibly because of certain conformational selections.

The fluctuation around D76 may affect other regions. Le Marchand et al. [15] also suggested that D76 is a “hub” of the network formed by some superficial residues including residues on the AB loop, around K41, around the mutation site, and the C-terminal region, and this network transmitted a change in dynamics. We speculated that the D-strand and DE loop regions are also involved in the dynamical linkage. Since K41 and D76 are located on the CD loop and EF loop, respectively, a change in the interactions of these residues may perturb the registration of hydrogen bonds formed between the D- and E-strands. As described above, the D-strand may assume two conformations: straight and bulged forms. These results indicate that the D-strand is also frustrated, and its conformation is affected by conformational changes around D76 through the interaction linkage. The disappearance of fluctuations following the application of pressure led to the specific ∆δ values of the affected residues (Figure 3E).

## 4. Materials and Methods

### 4.1. Expression and Purification of Wild-Type and D76N β_2_ms

A plasmid for the expression of the D76N β_2_m, pAED4/β_2_m D76N, was constructed by introducing a site-directed mutation into pHikaru [31], which is a pAED4 plasmid containing the human β_2_m gene, using the KOD plus mutagenesis protocol (Toyobo, Osaka, Japan). ^15^N-labeled wild-type and D76N β_2_ms were expressed in *Escherichia coli* strain BL21(DE3) (Novagen, Madison, WI, USA) and purified as described previously [31].

### 4.2. NMR Measurements

NMR measurements were performed using an AVANCE III-600 spectrometer equipped with a Z-axis gradient and triple resonance TXI probe (BrukerBioSpin Co., Rheinstetten, Germany) NMR data were processed using nmrPipe [32]. Signal intensities and chemical shifts were calculated using Sparky software (Goddard and Kneller, SPARKY 3, University of California, San Francisco, California, CA, USA). Subsequent numerical analyses were performed using Igor Pro (version 6.1.2.2, Wavemetrics, Lake Oswego, OR, USA).

Chemical shifts of signals in the ^1^H–^15^N HSQC spectrums for D76N were assigned based on information on the wild type. NOESY-HSQC and TOCSY-HSQC measurements were also performed for the confirmation of signal assignments.

Apparent chemical shift differences (∆δ_app_) were calculated using Equation (2):

(2) $$∆\delta_{\mathrm{app}}={\left(∆\delta_{H}{}^{2}+{\left(∆\delta_{N}/5\right)}^{2}\right)}^{0.5}$$

where ∆δ_H_ and ∆δ_N_ are chemical shift differences in the ^1^H and ^15^N axis directions, respectively, on HSQC spectra.

### 4.3. High-Pressure NMR Measurements

The β_2_m sample for high-pressure NMR was dissolved in 20 mM NaAc (pH 5.0 for WT and pH 5.5 for D76N) containing 10% D_2_O. The protein concentration was 8.5 mg/mL for WT and 4.0 mg/mL for D76N. High-pressure NMR measurements were performed at pressures ranging between 5 and 225 MPa using a homemade ceramic cell connected on-line to an external pump [33]. To avoid bubble formation, we maintained the lowest pressure at 5 MPa, instead of at 0.1 MPa.

To extract information on pressure-induced thermodynamic transitions from the ∆δ data obtained, we performed a PCA-based analysis [25]. The chemical shift data, δ_H_ and δ_N_ values, from each spectrum were represented as a single-row vector. Vectors were assembled into a 2D matrix **X**, in which the rows were chemical shift data from individual residues and the column vectors were the spectra at each pressure. The matrix size was 164 rows (82 (number of traceable residues) × 2 (δ_H_ and δ_N_)) × 10 columns (different pressure points) for both WT and D76N. The matrix **X** was subjected to a singular value decomposition and subsequent analyses. The procedure of the analysis was described in Supporting Information Figures S1–S3 of Sakurai and Goto [24]. Details on the process to obtain the present data are described in Supplementary Materials (Figure S4 and its caption).

### 4.4. Hydrogen/Deuterium (H/D) Exchange Experiment

H/D exchange experiments were performed at 25 °C and pD_reading_ 4.6 for WT and 5.1 for D76N by dissolving 2 mg (WT) or 5 mg (D76N) of lyophilized proteins into 500 μL of 20 mM NaAc prepared with D_2_O. The reaction was monitored by recording a series of ^1^H–^15^N HSQC spectra within one day. The exchange rate constant (*k*_ex_) of individual residues was analyzed using Sparky, assuming single exponential decay. Protection factors were calculated as a ratio between the observed rate constant (*k*_ex_) and the intrinsic exchange rate constant (*k*_int_) predicted from the amino acid sequence considering the experimental pH [34]. In the case of D76N, the formation of aggregates also simultaneously proceeded during the H/D exchange experiment, as revealed by a decrease in methyl proton signal intensities in 1D proton spectra. Thus, *k*_ex_ for D76N was calculated by *k*_ex_ = *k*_obs_/*k*_agg_, where *k*_obs_ and *k*_agg_ are the rate constants for the observed signal decays in the 2D ^1^H–^15^N and 1D methyl signals, respectively.

### 4.5. R_2_ Measurement

Measurements of the *R*_2_ values of ^15^N nuclei in backbone amides were performed using the pulse sequence described by Farrow et al. [35]. The experiment includes a series of 7 experiments with transverse decay times ranging between 17.6 and 228.8 ms.

### 4.6. MD Simulation

Experimentally obtained atomic coordinate files, 2yxf for wild-type β_2_m and 4fxl for the D76N mutant, have been downloaded from the Protein Data Bank Japan (PDBj) [36]. Missing hydrogen atoms were complemented by the pdb2gmx program in GROMACS programs under pH 7. The structure was immersed into a rectangular box of solvents composed of water molecules and sodium and chloride ions at concentrations of 150 mM. The minimum distance between the surfaces of the box and the atoms in the protein was set to 18 Å. The Amber force field [37] was applied to protein and the TIP3P model [38] to water. We treated the system under periodic boundary conditions. Electrostatic energy and force were calculated by the particle mesh Ewald method [39], with the cut-off length of van-der-Waals set to 8 Å. The system was minimized in total energy using the steepest decent method. Furthermore, chemical bonds associated with hydrogen atoms were constrained by the LINCS algorithm [40] and water molecules were rigidified by the SETTLE algorithm [41]. The energy minimized system was equilibrated using the MD simulation with the Berendsen algorithm [42] at 310 K. Furthermore, the MD simulation of the thermally equilibrated system was conducted under constant temperature (310 K) and pressure (both 0.1 and 200 MPa) conditions using the Parrinello–Rahman method [43]. The time step length of the MD simulations was set to 2 fs. A production run of the system was performed, and snap shots were collected every 2 ps for the analysis. All simulations were done by GROMACS programs [44].

## 5. Conclusions

In order to elucidate the destabilization mechanism of the D76N mutation for β_2_m, NMR experiments and MD simulations were performed. NMR measurements detected changes in residue-specific conformational changes upon the mutation or application of pressure. The MD simulation reproduced behaviors that were consistent to NMR results and provided more detailed insights into conformational changes. Correlated dynamics, which were observed in peripheral regions, including the mutation site and D-strand, conferred rigidness to inter-sheet packing. The D76N mutation attenuated the correlated motions and the subsequent loosening of inter-sheet packing. A key result was that the pressure perturbation mimicked the effects of the D76N mutation. Under these conditions, the application of pressure enabled us to observe continuous changes in dynamics and structures, thereby allowing us to characterize the mutation effect in more detail. Thus, pressure perturbation may be a distinct method for elucidating other mutation-induced structural and dynamic changes.

## Supplementary Materials

The following are available online at https://www.mdpi.com/2218-273X/9/9/491/s1, Figure S1: Displacement of C^α^ atoms between several crystal structures of β_2_m, Figure S2: Selection of experimental pH conditions for wild-type and D76N β_2_ms using tryptophan fluorescence, Figure S3: Comparisons of ∆δ_app_ values obtained for WT at pH 5.0 and 5.5, and D76N at pH 5.5, Figure S4: The data process of pressure-induced ∆δ data using the PCA-based method, Figure S5: The time courses of root mean square deviations of the MD structures, Figure S6: Positions of the side chains of Leu23 and Phe70.
