# Supplementary material for: Loosening of Side-Chain Packing Associated with Perturbations in Peripheral Dynamics Induced by the D76N Mutation of β2-Microglobulin Revealed by Pressure-NMR and Molecular Dynamic Simulations
Source: Biomolecules. 2019 Sep 16;9(9):491. doi: 10.3390/biom9090491 (PMC6769805; doi:10.3390/biom9090491)
Supplement: Supplementary file 1 [file biomolecules-09-00491-s001.pdf]

Supplementary Materials for

Loosening of side-chain packing associated with perturbations in peripheral dynamics induced by the D76N mutation of β2-microglobulin revealed by pressure-NMR and molecular dynamic simulations

Kazumasa Sakurai[1,*], Ryosuke Tomiyama[2], Takuma Shiraki[3], and Yasushige Yonezawa[1]

[1] High Pressure Protein Research Center, Institute of Advanced Technology, Kindai University, 930 Nishimitani, Kinokawa, Wakayama 649-6493, Japan

[2] Graduate School of Biology-Oriented Science and Technology, Kindai University, Wakayama 649-6493, Japan

[3] Department of Science and Technology on Food Safety, Faculty of biology-oriented science and technology, Kindai University, Wakayama 649-6493, Japan

* Correspondence: sakurai@waka.kindai.ac.jp; phone: +81-736-77-0345 (ex. 5004)

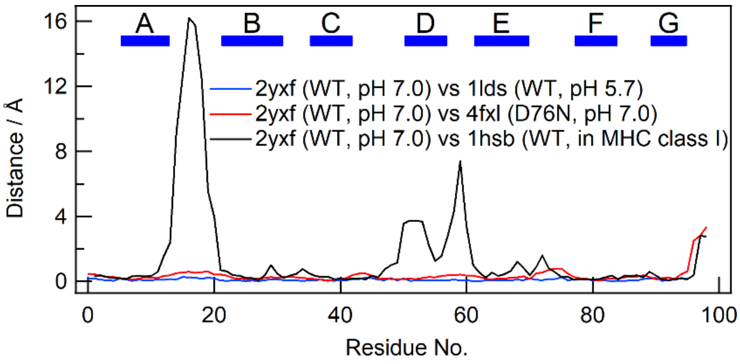

Figure S1. Displacement of C$^{\alpha}$ atoms between several crystal structures of β2m. The regions of residues 4–11, 21–40, 46–48, and 61–94 were aligned optimally and the distances between the same C$^{\alpha}$ atoms were then calculated. The PDB data examined were 2yxf (separate monomer, pH 7.0), 1lds (separate monomer, pH 5.7), 4fxl (D76N, pH 7.0), and 1hsb (X-ray, complexed state in MHC class 1).

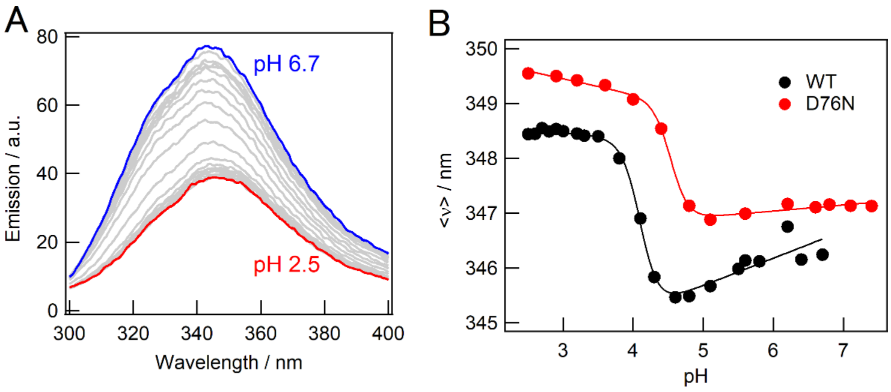

Figure S2. Selection of experimental pH conditions for wild-type and D76N β2ms using tryptophan fluorescence. (A) pH-dependent spectral change in the tryptophan fluorescence of wild-type β2m. (B) pH-dependent <ν> values for the wild type (black) and D76N β2m (red). Continuous lines indicate sigmoidal curves fit for the midpoint pH of unfoldings.

Selection of experimental pH conditions for wild-type and D76N β2ms using tryptophan fluorescence

pH-dependent spectral changes in the tryptophan fluorescence of wild-type and D76N β2ms were investigated (Figure S2A). The sample for measurement contained 0.05 mg ml^{-1} protein, 2 mM sodium phosphate, 2 mM sodium acetate, and 100 mM NaCl at 25°C. Excitation and emission wavelengths were 280 nm with a slit-width of 5 nm and 300-400 nm with a slit-width of 5 nm, respectively. The pH of 3 mL of the sample solution in a 1×1 cm quartz cell was lowered by gradually adding 1 N HCl solution and spectra were obtained with a FP-6500 spectrofluorometer (JASCO Inc., Japan) at the respective pH points. The starting pH value was 6.7. Red and blue lines in Figure S2A indicate the spectra obtained at pH 6.7 and 2.5, respectively. The spectra obtained were quantified using the center of the spectral mass, <ν>, which expresses the average energy of each spectrum calculated from the following equation:[1]

$$\langle \nu \rangle = \sum \nu_i F_i / \sum F_i$$

where ν_i and F_i are the wavelength and fluorescence intensity at ν_i. pH-dependent <ν> values (Figure S2B) were analyzed with a sigmoidal function to obtain the midpoint pH value of unfolding. The midpoint pH values of unfolding were 4.11 ± 0.04 and 4.54 ± 0.03 for the wild type and D76N, respectively.

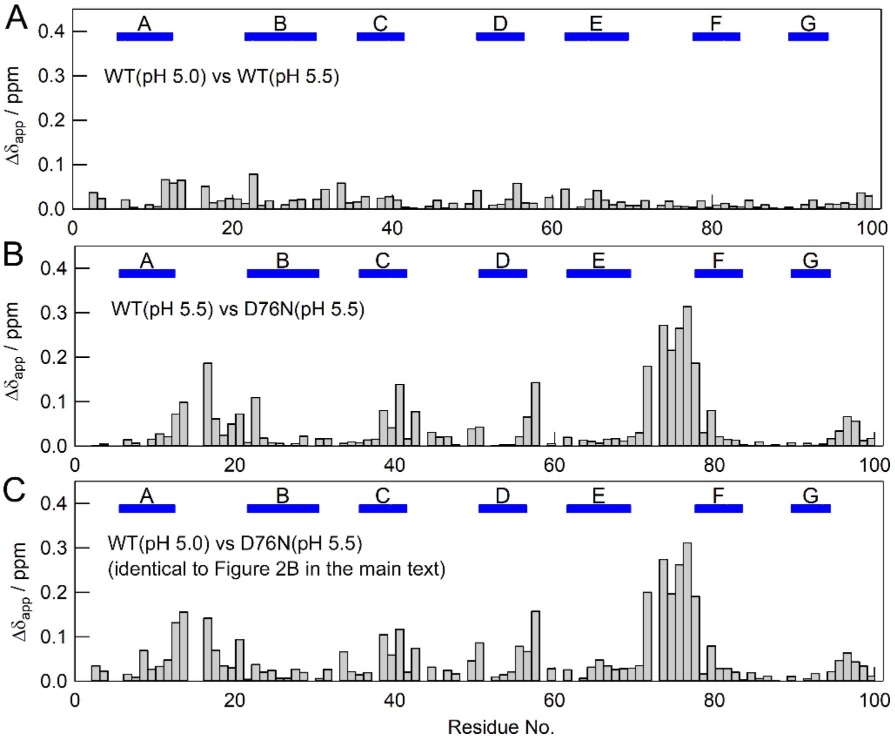

Figure S3. Comparisons of $\Delta\delta_{app}$ values obtained for WT at pH 5.0 and 5.5, and D76N at pH 5.5. We prepared a wild-type β_2m solution in 20 mM NaAc (pH 5.5). Then, measured the HSQC spectrum of the sample. Panel A shows the $\Delta\delta_{app}$ pattern between WT at pH 5.0 and pH 5.5. Panel B shows the $\Delta\delta_{app}$ pattern between WT and D76N at pH 5.5. Panel C (identical to the Figure 2B in the main text) shows the $\Delta\delta_{app}$ pattern between WT at pH 5.0 and D76N at pH 5.5. It was found that no significant $\Delta\delta_{app}$ values were observed for all residues in panel A. On the other hand, the $\Delta\delta_{app}$ patterns in panel B and C were similar to each other. These observations indicate that the $\Delta\delta_{app}$ pattern in Figure 2B reflects the conformational changes upon the mutation and does not include effects from the pH difference.

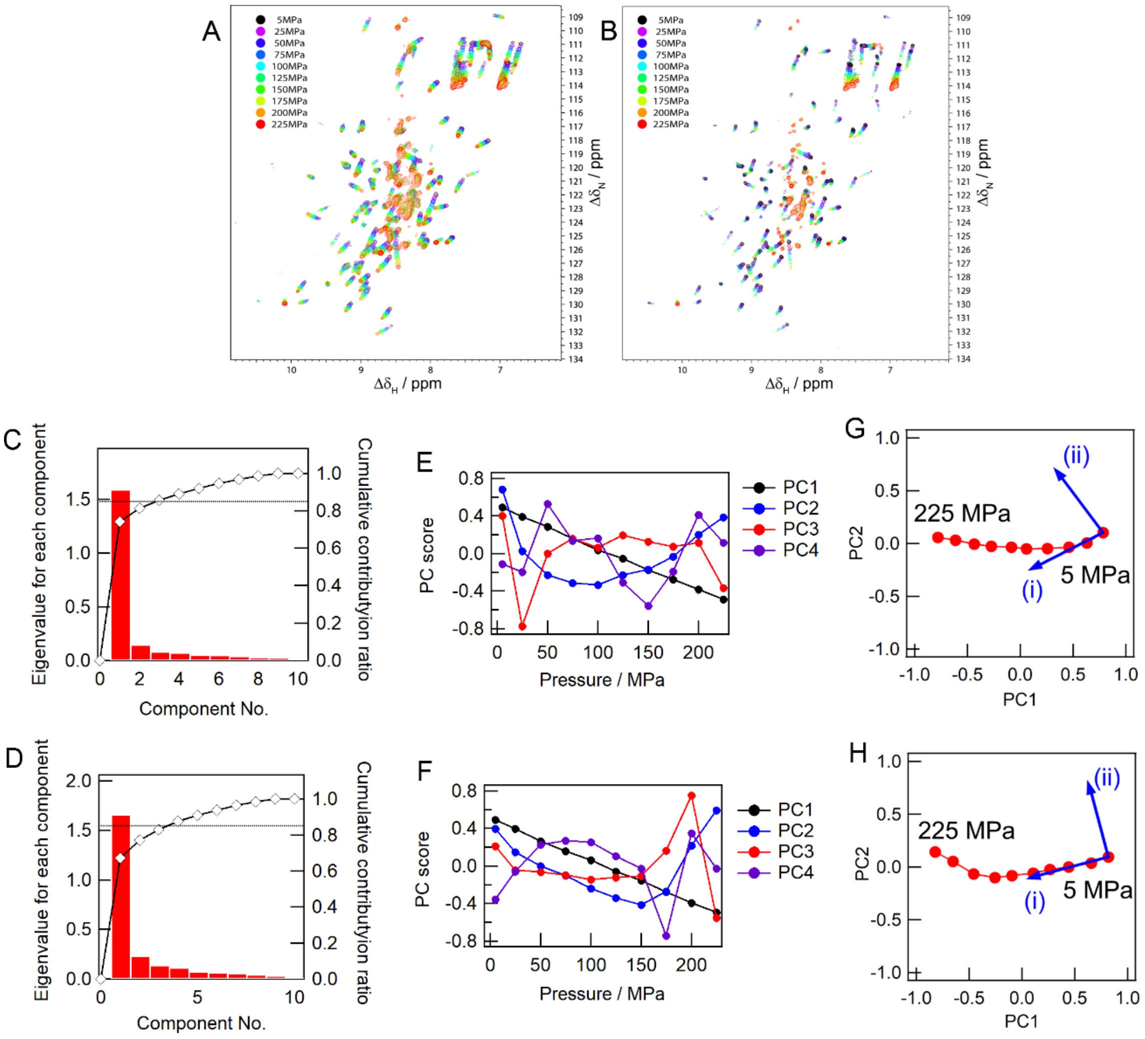

Figure S4. The data process of pressure-induced Δδ data using the PCA-based method. (A,B) Overlay of ^{1}H-^{15}N HSQC spectra of the wild type (A) and D76N β2m (B) obtained at pressures ranging between 5 and 225 MPa. The spectral color indicates the hydrostatic pressures at which measurements were performed. (C-F) Consideration of the significance of each PC obtained after SVD of the present Δδ data. The fractions of singular values of each component (red bars) and cumulative contribution ratio (black markers) for WT (C) and D76N (D), respectively. Scores obtained for the first four PCs as a function of pressure for WT (E) and D76N (F), respectively. The black, blue, red, and purple markers are the scores obtained for the first, second, third, and fourth PCs, respectively. (G,H) The PC planes of the pressure-dependent spectral changes for WT (G, identical to Figure 3B in the main text) and D76N (H, identical to Figure 3C) obtained from the SVD procedure. A point on the plane corresponds to one spectrum. The

rightmost point corresponds to the spectrum obtained in the initial conditions (5MPa). The blue arrows indicate the supposed directions of the conformational changes corresponding to (i) mechanical compression and (ii) thermodynamic transition.

The data process of pressure-induced Δδ data using the PCA-based method

We performed high-pressure NMR measurements for the wild type (Figure S4A). We conducted the singular value decomposition (SVD) of the pressure-dependent chemical shift data to obtain nine PCs and corresponding contribution ratios. The cumulative contribution ratio of the first two PCs was 0.81 (Figure S4C). Furthermore, the score plots, or variations in the fractions of each PC, of the 1st and 2nd PCs for all proteins were found to change smoothly in a pressure-dependent manner, whereas those of the 3rd PC showed abrupt changes at certain pressure points (Figure S4E). Therefore, we speculated that the first two PCs contained information on pressure-dependent structural changes, whereas the 3rd and higher PCs reflected the measurement noises of the chemical shift values. Figure S4G (identical to Figure 3B in the main text) shows a plot of PC1 and PC2. Although the major spectral change is in the direction of PC1, a significant contribution of PC2 was observed, which indicates that the spectral change includes contributions from two certain transitions, presumably (i) mechanical compression and (ii) thermodynamic transition.[2,3] The spectral change at lower pressure regions is often attributed to mechanical compression in the case of a structured protein. Thus, we set the direction along which the initial spectral change occurred as (i) mechanical compression. On the other hand, (ii) thermodynamic transition generally occurs in a certain pressure range. Thus, the direction of the spectral change observed in the middle pressure region is considered to represent thermodynamic transition (ii) and we assumed that its contributions may be orthogonal to the direction corresponding to (i) mechanical compression. Based on the directions identified, we calculated the Δδ patterns corresponding to the directions of vectors (i) and (ii) (Figure 3D and E in the main text). The same measurements and analyses were performed on D76N (Figure S4B). The cumulative contribution ratio of the first two PCs was 0.77 and we similarly interpreted the data obtained for D76N (Figure S4D,F). Therefore, we also discuss the data containing the first two PCs for D76N (Figure S4H and Figure 3C in the main text). The Δδ patterns obtained for (i) and (ii) on D76N are shown in Figure 3F and G in the main text, respectively.

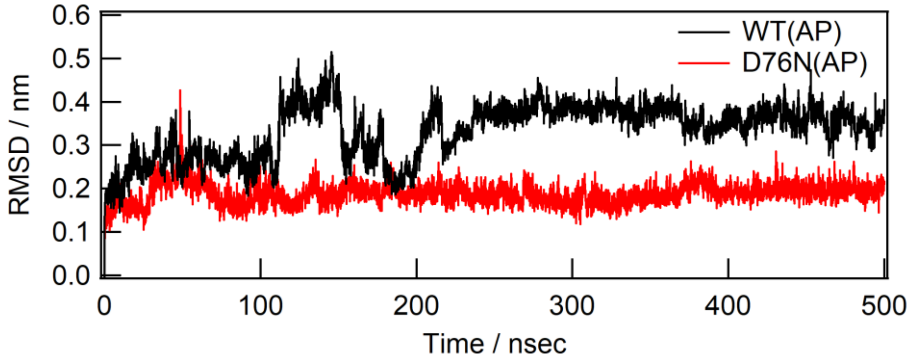

Figure S5. The time courses of the root mean square deviations for the main-chain C^{α} atoms of WT (black) and D76N (red) under pressure conditions of 0.1 MPa.

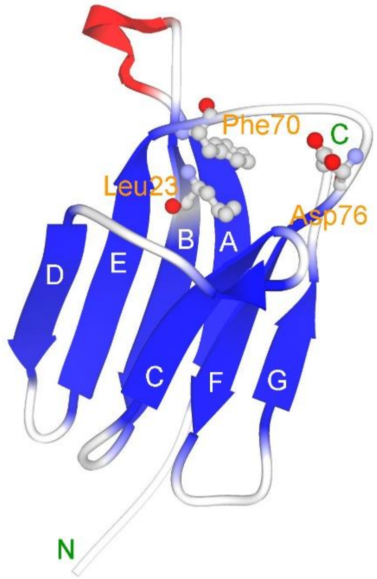

Figure S6. Positions of the side chains of Leu23 and Phe70. Crystal structure of β2m (PDB ID: 2yxf) is presented in a ribbon diagram. The green letters indicate the N- and C-terminal ends. The side chains of Leu23, Phe70, and Asp76 are depicted as balls and sticks.

References in Supplementary Materials

1. Silva, J. L.; Miles, E. W.; Weber, G. Pressure dissociation and conformational drift of the beta dimer of tryptophan synthase. *Biochemistry* **1986**, *25*, 5780-5786.
2. Kitahara, R.; Hata, K.; Li, H.; Williamson, M. P.; Akasaka, K. Pressure-induced chemical shifts as probes for conformational fluctuations in proteins. *Prog. Nucl. Magn. Reson. Spectrosc.* **2013**, *71*, 35-58.
3. Sakurai, K.; Maeno, A.; Lee, Y. H.; Akasaka, K. Conformational Properties Relevant to the Amyloidogenicity of beta2-Microglobulin Analyzed Using Pressure- and Salt-Dependent Chemical Shift Data. *J. Phys. Chem. B* **2019**, *123*, 836-844.
